# Improving the power for detecting overlapping genes from multiple DNA microarray-derived gene lists

**DOI:** 10.1186/1471-2105-9-S6-S14

**Published:** 2008-05-28

**Authors:** Xutao Deng, Jun Xu, Charles Wang

**Affiliations:** 1 Transcriptional Genomics Core, Burns Allen Research Institute, Cedars-Sinai Medical Center, Los Angeles, CA, USA; 2 Department of Medicine, UCLA David Geffen School of Medicine, Los Angeles, CA, USA

## Abstract

**Background:**

In DNA microarray gene expression profiling studies, a fundamental task is to extract statistically significant genes that meet certain research hypothesis. Currently, Venn diagram is a frequently used method for identifying overlapping genes that meet the investigator's research hypotheses. However this simple operation of intersecting multiple gene lists, known as the Intersection-Union Tests (IUTs), is performed without knowing the incurred changes in Type 1 error rate and can lead to loss of discovery power.

**Results:**

We developed an IUT adjustment procedure, called Relaxed IUT (RIUT), which is proved to be less conservative and more powerful for intersecting independent tests than the traditional Venn diagram approach. The advantage of the RIUT procedure over traditional IUT is demonstrated by empirical Monte-Carlo simulation and two real toxicogenomic gene expression case studies. Notably, the enhanced power of RIUT enables it to identify overlapping gene sets leading to identification of certain known related pathways which were not detected using the traditional IUT method.

**Conclusion:**

We showed that traditional IUT via a Venn diagram is generally conservative, which may lead to loss discovery power in DNA microarray studies. RIUT is proved to be a more powerful alternative for performing IUTs in identifying overlapping genes from multiple gene lists derived from microarray gene expression profiling.

## Background

Nowadays many microarray-based studies adopt complex experimental design involving multiple treatments, cell lines/tissues, multiple dosages, time points, phenotypes and so on [1-4]. These studies are often involved with complex research hypotheses. For instance in one of our previous studies [1], we were interested in identifying differentially expressed genes (DEGs) in responding to common bile duct ligation in bone marrow stem cells (BMSCs) compared against primary hepatocytes. Two DEG sets from BMSCs and hepatocytes were identified respectively, and the overlapping genes across the two cell types were obtained. The overlapping genes produced across the two cell types allowed the identification of common biological pathways, ontological classes, and biological mechanisms across the two cell types in responding to the treatment.

The intersection operations on multiple gene lists are equivalent to performing multiple tests for the combined hypotheses on every single gene. Although there are many statistical tests proposed for gene expression studies [5-8], the problems of obtaining overlapping gene sets based on multiple tests were overlooked in microarray-based studies. To obtain genes that satisfy the specific hypotheses, researchers simply overlap the gene sets from multiple gene sets and visualized them in Venn diagrams. However, because of lacking multiplicity adjustment, this procedure overlooks the changes of statistical properties, i.e., power, type 1 error rate, p-values, during the intersection operations. This type of multiple testing for finding overlapping genes is known as the Intersection-Union Test (IUT). Despite some early efforts [9-11], the statistical properties and adjustment algorithms of IUT are not well established. Berger has proved that IUT without multiplicity adjustment is a level-α test [10], when the individual tests were controlled at type 1 error rate α. However, the family wise error rate (FWER) for IUT α' is generally much smaller than α. Therefore, performing IUT without multiplicity adjustment would be very conservative and result in too many false negatives.

In this paper, we show that current overlapping operation, applying no p-value adjustment for IUT, is overly conservative in general. As a result, current microarray studies suffer from low power in detecting overlapping genes and therefore limit its use in biological data mining. We developed an analytical solution, named as Relaxed IUT (RIUT) for the multiplicity adjustment of IUTs under certain conditions. We theoretically proved that our proposed method is a less conservative and more powerful than current approaches. We demonstrated the superiority of RIUT for detecting overlapping genes in simulated data sets and complex microarray-based toxicogenomic studies.

## Results

### Monte-Carlo simulation results of RIUT

As an example to showcase the power of RIUT, the mRNA expression of a given gene is tested whether it is significantly altered by a drug treatment in multiple tissues. Suppose gene expressions were measured in *m *different tissues and one is interested in the overlapping DEGs. For each tissue, a two-sample t-test is performed between a treatment group and a control group, each containing *n *replicates to obtain a list of significant genes for that tissue. Then we have an IUT that is constructed by *m *individual tests, each for a different tissue:

*H*_0*i*_: the drug has no effect in the *i*th tissue, i.e., μ_*ti *_= μ_*ci*_,

*H*_*Ai*_: the drug has effect in the *i*th tissue, i.e., μ_*ti *_≠ μ_*ci*_, 1 ≤ *i *≤ *m*,

where μ_*ti *_and μ_*ci *_denote the expression mean of the treatment and the control groups respectively of the *i*th tissue. The hypotheses for IUT are *H*_0_: the drug shows no effect on at least one tissue vs. *H*_*A*_: the drug shows effect on all tissues.

For the Monte-Carlo simulation, the expression data for the treatment and control groups were modeled as normal distributions *N*(μ_*ti*_, 1) and *N*(μ_*ci*_, 1) respectively, where μ_*ci *_= 0, 1 ≤ *i *≤ *m*. We then drew *n *= 5 samples from each of these distributions and apply RIUT and BIUT to these simulated data. Table 1 shows the estimated type 1 error rate by 10000 simulated instances of IUT formed by 2 individual tests. Expression mean μ_*t*1 _is fixed at 0 for the first tissue and different values of μ_*t*2 _were used to represent the drug having diverse effects (μ_*t*2 _= 0, 0.5, ..., 4.5, 5.0) on the second tissue. Overall, the drug has no effect on the first tissue and the null hypothesis *H*_0 _of IUT is true. Results show that both RIUT and traditional Berger's IUT (BIUT) are bounded by nominal α at which the individual hypotheses were tested. The actual type 1 error for IUT is generally smaller than α. RIUT is less conservative than BIUT as it achieves a type 1 error rate that is closer to α. To prove concept, we also tested two meta-analysis methods for combining independent tests, the Fisher's method [15] and Stouffer's method [16]. The results show that their actual type 1 rate can be so much higher than the nominal one that these methods are not suitable for the IUTs.

**Table 1:** Monte-Carlo estimates of type 1 error rate *α*^'^(%)

*μ*_*t*2_	RIUT	BIUT	Fisher	Stouffer
0.0	0.047	0.003	0.050	0.050
0.5	0.044	0.006	0.090	0.085
1.0	0.035	0.015	0.210	0.188
1.5	0.035	0.027	0.405	0.327
2.0	0.040	0.039	0.632	0.476
2.5	0.049	0.048	0.821	0.621
3.0	0.048	0.048	0.931	0.729
3.5	0.047	0.047	0.982	0.808
4.0	0.051	0.051	0.995	0.875
4.5	0.048	0.048	0.999	0.907
5.0	0.046	0.046	1.000	0.930

*m *= 2, *n *= 5, and *μ*_*t*1 _= 0.0; individual tests were performed at *α *= 0.05 and each estimate were based on 10000 runs.

**Figure 1:**
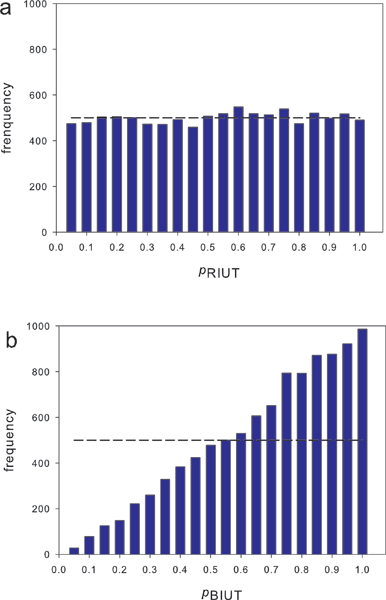
**Distributions of the p-values of 10000 simulations with μ_*t*1 _= μ_*t*2 _= 0.0 using RIUT and BIUT**. a. The distribution of RIUT p-value *p*'. b. The distribution of unadjusted BIUT p-value *p*. The dashed line indicates the hypothetical uniform distribution of the p-values of the 10000 runs.

At μ_*t*1_= μ_*t*2 _= 0, the resultant p-value distributions generated from 10000 instances were illustrated in Figure 1. Theoretically, the p-values originated from null hypothesis should appear approximately uniformly distributed (the dashed line). The adjusted *p' *using RIUT achieved the desired distribution as shown in Figure 1a. However, Figure 1b shows that the unadjusted *p *is seriously skewed to the right, indicating that the test is overly conservative.

Table 2 shows the power estimate of IUT consisting of two individual tests and both individual null hypotheses are not true (μ_*t*1 _= 0.5, μ_*t*2 _= 0, 0.5, ..., 4.5, 5.0). RIUT demonstrated higher power than BIUT at small and moderate effect size. At large effect size, RIUT and BIUT show essentially the same power.

**Table 2:** Monte-Carlo estimates of power (%)

*μ*_*t*2_	*α *= 0.05		*α *= 0.01	
	
	RIUT	BIUT	RIUT	BIUT
0.5	0.057	0.014	0.007	0.001
1.0	0.059	0.029	0.006	0.003
1.5	0.077	0.064	0.008	0.006
2.0	0.085	0.081	0.014	0.012
2.5	0.101	0.101	0.020	0.020
3.0	0.103	0.103	0.023	0.023
3.5	0.109	0.109	0.030	0.030
4.0	0.105	0.105	0.024	0.024
4.5	0.112	0.112	0.027	0.027
5.0	0.104	0.104	0.024	0.024

*m *= 2, *n *= 5, and *μ*_*t*1 _= 0.5; individual tests were performed at *α *= 0.05 or *α *= 0.01 and each estimate based on 10000 runs.

As a more realistic simulation, we pooled instances being positive (*H*_0 _not true) and negative (*H*_0 _true). We use γ to denote the simulation Bernoulli probability that an individual hypothesis is not null such that Prob(*H*_*Ai*_) = γ. The population means were set at μ_*t*1 _= μ_*t*2 _= 0.5. According to this procedure, we simulated 10000 instances under different γ and the resultant number of true negative and true positive were denoted as *K*_0 _and *K*_1 _respectively. The identified false positive and true positives were notated as *V *and *S *respectively. The results in Table 3 confirmed our previous observations that RIUT is a more powerful and less conservative. The same patterns were observed in simulations using other parameter values (results not shown).

**Table 3:** Simulation results of pooled instances

*γ*	*K*_0_	*K*_1_	RIUT		BIUT	
			
			*V *(type 1)	*S*(power)	*V *(type 1)	*S(power)*
0.1	9899	101	482(0.049)	12(0.119)	24(0.002)	1(0.010)
0.2	9607	393	447(0.047)	39(0.099)	32(0.003)	6(0.015)
0.3	9087	913	394(0.043)	98(0.107)	29(0.003)	8(0.009)
0.4	8427	1573	363(0.043)	126(0.080)	33(0.004)	21(0.013)
0.5	7560	2440	310(0.041)	202(0.083)	30(0.004)	28(0.011)
0.6	6470	3530	270(0.042)	264(0.075)	22(0.003)	35(0.010)
0.7	5023	4977	209(0.042)	317(0.064)	23(0.005)	59(0.012)
0.8	3534	6466	119(0.034)	410(0.063)	10(0.003)	83(0.013)
0.9	1893	8107	75(0.040)	483(0.060)	10(0.005)	84(0.010)

*m *= 2, *n *= 5, and *μ*_*t*1 _= *μ*_*t*2 _= 0.5; individual tests were performed at *α *= 0.05 and each estimate were based on 10000 runs.

### Identifying overlapping genes that respond to multiple drug treatment

This example illustrates how RIUT algorithm can be used in real DNA microarray-based multiple-testing problems. Originally generated from the MAQC project [2], this data set consists of rat RNA samples that came from six treatment/tissue groups. The treatment/tissue groups were aristolochic acid/liver, aristolochic acid/kidney, riddelliine/liver, comfrey/liver, control/liver and control/kidney. There were six biological replicates in each treatment/tissue group. mRNA expression profiles were obtained using four commercial platforms including Affymetrix (Rat Genome 230 2.0), Agilent (Whole Rat Genome Oligo Microarray, G4131A), Applied Biosystems (Rat Genome Survey Microarray) and GE Healthcare (RatWhole Genome Bioarray, 300031) in five different labs with two labs using the Affymetrix microarray platform. Totally, 180 chips were obtained and the cross-platform probe-mapping gave rise to 4609 genes commonly detected across four platforms.

In this example, our goal is to identify the common genes responding to different drug treatments. These genes may shed lights on common cytotoxicity mechanisms of these drugs. We used the liver/control (L_CTL) group as control and the 3 drugs treatment at rat liver were referred to as L_AA, L_CFY, and L_RDL respectively. The IUT consists of two individual tests, (i.e., *t*_1_: L_AA vs. L_CTL and *t*_2_: L_CFY vs. L_CTL), each testing whether a gene differentially expressed in response to one specific drug treatment versus control. The combined IUT was used to identify the genes that differentially expressed in combined each pair of the three treatments. Table 4 shows the number of overlapping differentially expressed genes identified using RIUT compared with the traditional BIUT at different labs/platforms. As expected, the number of significant genes obtained using RIUT is consistently greater than that obtained using BIUT. The magnitude of increase ranges from 13% to 184%. For many IUTs, the number of identified overlapping genes using traditional BIUT is close to the number of nominal false positives (230), suggesting that BIUT lacks power to identify true overlapping differentially expressed genes. The testing results using real microarray data confirm our analytical results of Theorem 1 and Theorem 2 (shown in Methods). In addition our Monte-Carlo simulation demonstrates that RIUT is a more powerful and less conservative approach than BIUT.

**Table 4:** Number of differentially expressed genes in all drug treatment groups

Platforms	IUT : A		IUT : B		IUT : C	
	
	RIUT	BIUT	RIUT	BIUT	RIUT	BIUT
Applied Biosystems	1160	1011	**1143** **13%**	**1008**	940	763
Agilent	362	262	525	413	452	159
GE Healthcare	697	528	862	723	639	415
Affymetrix (Site 1)	359	251	502	382	**422** **184%**	**175**
Affymetrix (Site 2)	524	375	724	556	521	289

*α *= 0.05 (Nominal # false positive < 230)

IUT A: t_1_: L_AA vs. L_CTL and t_2_: L_CFY vs. L_CTL

IUT B: t_1_: L_RDL vs. L_CTL and t_2_: L_CFY vs. L_CTL

IUT C: t_1_: L_AA vs. L_CTL and t_2_: L_RDL vs. L_CTL

### Detecting genes with time-course and dose-response effect to chemical treatment

To further demonstrate the applicability of RIUT in microarray studies, we focus on the rat cadmium toxicogenomic data set [3,18]. This study employed a more complex study design, in which both gene expression and cytotoxicity changes were profiled in a multi-dose multi-time-point setting. Briefly, primary rat hepatocytes were isolated and were exposed to three different doses of cadmium acetate (0, 1.25 and 2.0 μM) for 2 h. Cells were collected at 0, 3, 6, 12 and 24 h in all three groups (0, 1.25 and 2.0 μM Cd) for cytotoxicity evaluation by lactase dehydrogenase (LDH) leakage as well as for mRNA expression profiling by DNA microarray. Affymetrix GeneChip^® ^oligonucleotide arrays (RatTox U34) were used for mRNA expression profiling. There are 972 probe sets representing ~800 important toxicology-related genes in the RT U34 array. The microarray experiment was repeated using primary hepatocytes from 3 animals, each with 2 replicates (independent cultures) for each dosage (3 dosage levels) at each time point (5 time points), resulting in a total of 90 chips (3 animals • 2 replicates • 3 doses • 5 time points). The 2 replicates were averaged in our analysis.

Firstly, to identify differentially expressed genes in responding to cadmium treatment at each time point, two-sample t-tests were performed between the treatment (1.25 and 2.0 μM Cd) and control at each time point. Secondly, to identify the genes with persistent differentially expression due to cadmium exposure across different time points, the overlapping of the DEGs at both short term (3 h) and long term (12 h) were identified using our proposed method. The second research question was then formulated an IUT problem which could be solved using our proposed method.

**Figure 2:**
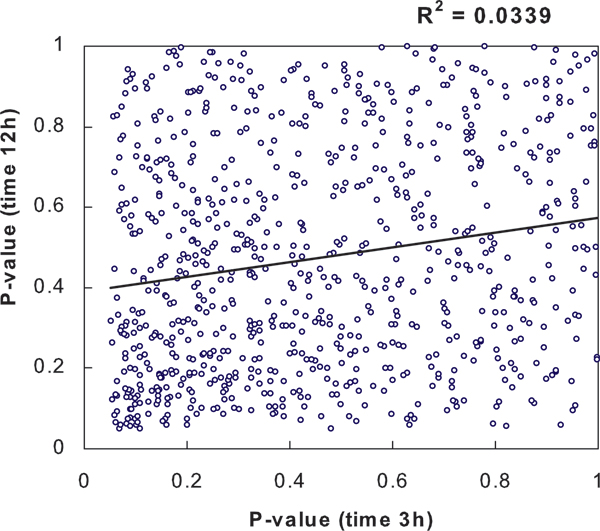
**The joint p-value distribution from *t*_1 _and *t*_2 _for all genes**. The IUT consists of two individual two-sample t tests (*t*_1_: 2.0 μM Cd vs. control at 3 h, *t*_2_: 2.0 μM Cd vs. control at 12 h).

We constructed an IUT consisting of two individual tests (*t*_1_: 2.0 μM Cd vs control at 3 h, and *t*_2_: 2.0 μM Cd vs control at 12 h). The joint p-value distribution from *t*_1 _and *t*_2 _for all genes is illustrated in Figure 2. It is shown that *t*_1 _and *t*_2 _were approximately independent (*R*^2 ^= 0.03) and their p-values were approximately uniformly distributed under null hypothesis (*p*_1 _> 0.05 and *p*_2 _> 0.05). Therefore the assumptions in Theorem 1 and 2 were not violated. In fact for most microarray-based studies, these assumptions need to be checked by scatter plot.

We performed RIUT and BIUT (*m *= 2, *n *= 3) for all 972 probe sets. RIUT identified 80 overlapping probe sets and traditional BIUT identified 19 at α = 0.05. The power of IUT methods directly affected the identified gene set on which the follow-up pathway interpretation was based. To demonstrate this effect, a pathway enrichment search was performed by comparing the IUT-identified gene sets with the specific KEGG pathways [19-21] using a Fisher's Exact Test on DAVID 2006 [22], resulting in an enrichment p-value for each pathway which were listed in Table 5. Using the RIUT gene set, we identified 3 significantly over-enriched pathways (Fisher Exact p-value < 0.05). However, using the BIUT gene set, no significant pathways in KEGG was identified. It has been reported that the *metabolism of xenobiotics by cytochrome p450 *pathway is significantly affected by cadmium exposure [23,24]. The activity of *MAPK signaling *pathway and *porphyrin and chlorophyll *metabolism pathway have also been affected by environmental cadmium exposure [25,26]. Again, Our analysis results using Cd time-course data set indicate that RIUT is a more powerful method than BIUT and the significance can be seen in the biological pathway identification.

**Table 5:** KEGG pathways Identified and the enrichment *p*-values

**KEGG Pathways**	**Fisher's Exact Test P-Value**
Metabolism of xenobiotics by cytochrome P450	0.004
MAPK signaling pathway	0.028
Porphyrin and chlorophyll metabolism	0.030

(Fisher's Exact Test) based on the gene sets derived from RIUT and BIUT

## Discussion

RIUT can be improved in three ways to be applicable in more scenarios: (1) estimating unknown nuisance parameters; (2) dealing with more than 2 individual tests; and (3) combining non-independent tests. For the scenario (1), the current approach for estimating π with an arbitrary λ was originally proposed by Storey [14] for estimating false discovery rate. The authors proposed a bootstrap method for finding optimal λ which can also be used here. It should be noted that Theorem 2 is valid no matter how the λ is chosen and how good π is estimated. For IUTs consisting of more than 2 individual tests, it is difficult to obtain the analytical solution as Theorem 1. However, we can apply a step-up procedure which agglomeratively applies RIUT on its least significant individual test. It is more difficult to extend our procedure for non-independent tests. It may need a resampling-based algorithm to incorporating correlation structure of multiple tests and dealing with non-normality issues. Resampling needs intensive computation which can be largely offset by today's powerful and inexpensive computing facility. Resampling of IUT is based on resampling of individual tests which can be conveniently performed by either bootstrap or permutation. Bootstrap and permutation were discussed in many literatures [12,27,28].

## Conclusion

Our study demonstrated that the current unadjusted IUT approaches were overly conservative, which resulted in loss of power in finding overlapping genes in microarray-based gene expression studies. Our proposed RIUT was analytically proved to be a more powerful and less conservative approach than the current unadjusted IUT. The power improvement is more apparent in tests with weak and moderate effect sizes. This is also demonstrated in Monte-Carlo simulations and real case studies. In addition, certain known biologically relevant pathways were identified using the RIUT-derived overlapping genes which were not detected by using the traditional BIUT.

## Appendix

Let ***X ***denote the random vector of data values. Suppose the probability distribution of ***X ***depends on an unknown parameter *θ*. The set of possible values for *θ *will be denoted by Θ. Suppose we have *m *individual tests and let *R*_*i *_denote a rejection region for a level-*α *test of *H*_0*i *_: *θ *∈ Θ_*i *_versus *H*_*Ai *_: *θ *∈ $\Theta_{i}^{c}$, 1 ≤ *i *≤ *m*, where Θ_*i *_is a specified subset of Θ and $\Theta_{i}^{c}$ is its complement. Then IUT tests the union of sets against an intersection of sets. $H_{0}:\theta \in \Theta_{0}=\cup_{i=1}^{m}\Theta_{i}$ versus $H_{A}:\theta \in \Theta_{0}^{c}=\cap_{i=1}^{m}\Theta_{i}^{c}$, with the rejection region $R=\cap_{i=1}^{m}R_{i}$. In other words, the IUT rejects only if all of the tests reject.

**Berger's Theorem: **IUT with rejection region *R *is a level-*α *test of *H*_0 _versus *H*_*A*_.

*Proof*. For any *θ *∈ Θ_0 _and for any 1 ≤ *l *≤ *m*, we have $P_{\theta }(R)=P_{\theta }(\cap_{i=1}^{m}R_{i})\leq P_{\theta }(R_{l})\leq \alpha$. Therefore, is IUT is a level-*α *test. ▪

## Methods

### IUT and UIT

Suppose a gene on which a number of α-level hypothesis tests were performed, represented as *t*_1_, *t*_2_,..., *t*_*m*_, where *m *is the number of individual tests. Each test *t*_*i *_tests the null hypothesis *H*_0*i *_versus alternative hypothesis *H*_*Ai*_. We can combine all tests into a Union-Intersection Test (UIT) which rejects if *any *of the *t*_*i *_rejects. We can also combine all tests into an Intersection-Union Test (IUT) which rejects if *all *the *t*_*i *_reject. The UIT tests the hypothesis *H*_0 _= {all *H*_0*i *_are true} against *H*_*A *_= {at least one *H*_0*i *_is false} and the IUT tests the hypothesis *H*_0 _= {at least one *H*_0*i *_is true} against *H*_*A *_= {all *H*_0*i *_are false}. IUT and UIT were named from the fact that their null and alternative hypothesis can be described by set intersections and unions. (see Appendix for details).

The FWER of UIT is defined as α' = Pr(Reject at least one *H*_0*i *_| all *H*_0*i *_are true). It is well known that in general α' ≠ α. For example, if α = 0.05 and *m *= 5, α' would be about 0.23 when all individual tests are independent. Therefore there is a need to adjust α' for IUT and there exist many procedures to do so, ranging from simple Bonferroni correction to computer-intensive resampling-based correction [12,13]. The FWER IUT is α' = Pr(Reject all *H*_0*i *_| at least one *H*_0*i *_is true). It is also obvious that α' ≠ α for IUT in general. However unlike the well studied UIT, there is no known procedure for adjusting the FWER α' for IUT. The unadjusted IUT, also known as the Berger's approach, denoted as BIUT, suggests that the overall unadjusted p-value for IUT is

(1) *p *= max *p*_*i*_, 1 ≤ *i *≤ *m*,

where *p*_*i *_is the p-value for individual tests *t*_*i*_. Berger proved [10] that the unadjusted IUT is a level-α test if all *t*_*i *_are *level*-α tests. Berger's also showed that the above IUT is a *size*-α test under certain trivial case such as the case when exactly one *H*_0*i *_is true while all the other *H*_0*i *_are false. However, the unadjusted approach is not a size-α test in general. For example, when considering two independent individual tests *t*_1 _and *t*_2_, the chance of rejecting both hypotheses is α^2 ^rather than α if both *H*_01 _and *H*_02 _are true. Nonetheless, due to its simplicity, the unadjusted IUT approach was implicitly adopted by current microarray studies when overlapping genes were taken from several significant gene lists. This BIUT is equivalent to the Venn diagram in obtaining overlapping genes from multiple significant gene lists.

### Exact solution for IUT consisting of independent tests

Berger's approach can be very conservative and therefore substantial power could be lost for detecting overlapping genes. Given the observed p-values (*p*_1_, *p*_2_,..., *p*_*m*_) for the *m *individual tests, we are interested in estimate p-value for the entire test *H*_0 _of IUT. Here, we define a Westfall-Young-style p-value [12] for IUT denoted as *p'*,

(2) $$\begin{array}{l} p'=\mathrm{Pr}(\text{all }P_{j}\leq p|H_{0})\\ \begin{array}{cc} =\mathrm{Pr}\left(\max P_{j}\leq \max p_{i}|H_{0}\right)\text{,} & 1\leq i,j\leq m, \end{array} \end{array}$$

where *P*_j _denote the distribution for the p-value of the *j*th hypothesis under null hypothesis for IUT *H*_0 _= {at least one *H*_0*i *_is true}. The least significant p-value *p *is an observed statistic and the random variable max *P*_*j *_is the test statistic under *H*_0_. This definition is intuitive as p-value measures the probability of false positive under null hypothesis, where a false positive of IUT means that all the p-values under *H*_0 _are less than the observed *p*. Similar to the above equation, we have the following relationship between the IUT FWER α', and individual test type 1 error rate α.

(3) *α*' = Pr(all *P*_*j *_≤ *α *|*H*_0_) 1 ≤ *i*, *j *≤ *m*

Unlike UIT, the null hypothesis *H*_0 _of IUT is a composite hypothesis and contains nuisance parameters. However under certain conditions, it is possible to derive the analytical solution for α'. We obtain the following theorems:

#### Theorem 1

For an IUT constructed from two independent tests *t*_1_: *H*_01 _vs. *H*_*A*1_, and *t*_2_: *H*_02 _vs. *H*_*A*2_, both at significance level α, suppose the observed p-values for *t*_1 _and *t*_2 _are uniformly distributed under *H*_01 _and *H*_02 _respectively, the exact FWER α' for the IUT is controlled at

(4) $$\alpha '=\frac{(1-\pi_{1})\pi_{2}(1-\beta_{2})\alpha +(1-\pi_{2})\pi_{1}(1-\beta_{1})\alpha +(1-\pi_{1})(1-\pi_{2})\alpha^{2}}{1-\pi_{1}\pi_{2}},$$

where the true probabilities of alternative hypotheses are Pr(*H*_*A*1_) = π1 and Pr(*H*_*A*2_) = π2; the type 2 error rate for *t*_1 _and *t*_2 _are β1 and β2.

Proof:

$$\begin{array}{l} \alpha '=\mathrm{Pr}(\text{ max(}P_{1}\text{ ,}P_{2})\leq \alpha |H_{0})\\ =\mathrm{Pr}(\;P_{1}\;\leq \alpha ,P_{2}\leq \alpha |H_{0})\\ =\frac{\mathrm{Pr}(\;P_{1}\;\leq \alpha ,P_{2}\leq \alpha \text{,}H_{0})}{\mathrm{Pr}(H_{0})}\\ =\frac{\mathrm{Pr}(\;P_{1}\;\leq \alpha ,P_{2}\leq \alpha ,\text{(}H_{01}\text{ or }H_{02}\text{)})}{\mathrm{Pr}(\;H_{01}\text{ or }H_{02})}\\ =\begin{array}{l} \mathrm{Pr}(\;P_{1}\;\leq \alpha ,P_{2}\leq \alpha ,H_{01},H_{A2})+\\ \mathrm{Pr}(\;P_{1}\;\leq \alpha ,P_{2}\leq \alpha ,H_{A1},H_{02})+\\ \mathrm{Pr}(\;P_{1}\;\leq \alpha ,P_{2}\leq \alpha ,H_{01},H_{02}) \end{array}/1-\mathrm{Pr}(\;H_{A1}\text{)Pr(}H_{02})\text{ independence}\\ =\frac{(1-\pi_{1})\pi_{2}(1-\beta_{2})\alpha +(1-\pi_{2})\pi_{1}(1-\beta_{1})\alpha +(1-\pi_{1})(1-\pi_{2})\alpha^{2}}{1-\pi_{1}\pi_{2}}\text{ uniform} \end{array}$$

▪

Recall that the p-value is the lowest level of significance at which the null hypothesis could have been rejected. We can obtain the adjusted p-value based on the observed p-values of the individual tests

(5) $$p'=\frac{(1-\pi_{1})\pi_{2}(1-\beta_{2})p+(1-\pi_{2})\pi_{1}(1-\beta_{1})p+(1-\pi_{1})(1-\pi_{2})p^{2}}{1-\pi_{1}\pi_{2}}$$

where *p *= max(*p*_1_, *p*_2_).

#### Theorem 2

The RIUT procedure is universally at least as powerful as the unadjusted IUT, such that *p' *≤ *p*.

Proof:

$$\begin{array}{l} p'\leq \frac{(1-\pi_{1})\pi_{2}p+(1-\pi_{2})\pi_{1}p+(1-\pi_{1})(1-\pi_{2})p^{2}}{1-\pi_{1}\pi_{2}}\\ \leq \frac{\left(1-\pi_{1})\pi_{2}+(1-\pi_{2})\pi_{1}+(1-\pi_{1})(1-\pi_{2}\right)}{1-\pi_{1}\pi_{2}}p\\ \leq p \end{array}$$

▪

**Figure 3:**
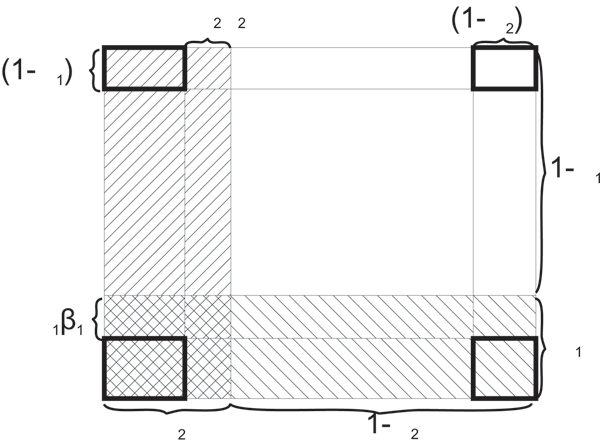
**Partition of the sample space of the two dimensional outcome space defined by *p*_1 _and *p*_2_**. The shaded two areas are the true non-null areas for the two individual tests respectively. The rejected regions by both tests are highlighted in bold rectangles at the four corners of the outcome space. Only the lower-left corner is the non-null region from both tests and therefore should be rejected by IUT. The other three corners are type 1 error region for IUT because at least one individual test is making type 1 error.

The above derivation can be visualized in Figure 3, which shows the partition of outcome space (*p*_1_, *p*_2_) of the two independent tests.

The relationship between α' and α is much more complicated in the IUT than that in the UIT. To apply the above theorem, the nuisance parameters (*π*_1_, *π*_2_, *β*_1_, *β*_2_) need to be estimated. Since there are usually thousands of genes available for each statistical test, we can obtain crude and conservative estimates of the parameters according to [14].

(6) $$\begin{array}{ccccc} {\hat{\pi }}_{i}(\lambda )=\frac{\#\{p_{i}(j)<\lambda \}}{(1-\lambda )n} & i=1,2, & j=1,...,n, & \text{and} & {\hat{\beta }}_{1}={\hat{\beta }}_{2}=0, \end{array}$$

where *n *is the total number of genes, *λ *is a chosen fixed value at 0.25 and *p*_*i*_(*j*) represents the observed *p*-value for the *i*th test on the *j*th gene.

## Competing interests

The authors declare that they have no competing interests.

## Authors' contributions

XD and CW conceived of the study. XD drafted the manuscript and implemented the algorithm and performed most of the analysis. JX helped in data acquisition and results interpretation. All authors revised and approved the paper.

## References

[B1] Wang C, Chelly MR, Chai N, Tan Y, Hui T, Li H (2005). Transcriptomic fingerprinting of bone marrow-derived hepatic beta2m-/Thy-1+ stem cells. Biochem Biophys Res Commun.

[B2] Guo L, Lobenhofer EK, Wang C, Shippy R, Harris SC, Zhang L (2006). Rat toxicogenomic study reveals analytical consistency across microarray platforms. Nat Biotechnol.

[B3] Tan Y, Shi L, Hussain SM, Xu J, Tong W, Frazier JM (2006). Integrating time-course microarray gene expression profiles with cytotoxicity for identification of biomarkers in primary rat hepatocytes exposed to cadmium. Bioinformatics.

[B4] Lim DA, Suarez-Farinas M, Naef F, Hacker CR, Menn B, Takebayashi H (2006). In vivo transcriptional profile analysis reveals RNA splicing and chromatin remodeling as prominent processes for adult neurogenesis. Mol Cell Neurosci.

[B5] Kooperberg C, Aragaki A, Strand AD, Olson JM (2005). Significance testing for small microarray experiments. Stat Med.

[B6] Wang A, Gehan EA (2005). Gene selection for microarray data analysis using principal component analysis. Stat Med.

[B7] Baldi P, Long AD (2001). A Bayesian framework for the analysis of microarray expression data: regularized t-test and statistical inferences of gene changes. Bioinformatics.

[B8] Tusher VG, Tibshirani R, Chu G (2001). Significance analysis of microarrays applied to the ionizing radiation response. Proc Natl Acad Sci USA.

[B9] Berger RL, Hsu JC (1996). Bioequivalence trials, intersection-union tests, and equivalence confidence sets. Statistical Science.

[B10] Berger RL (1982). Multiparameter hypothesis testing and acceptance sampling. Technometrics.

[B11] Allison DB, Cui X, Page GP, Sabripour M (2006). Microarray data analysis: from disarray to consolidation and consensus. Nat Rev Genet.

[B12] Westfall PH, Young SS (1993). Resampling-Based Multiple Testing: Examples and Methods for p-Value Adjustment.

[B13] Miller RGJ (1991). Simultaneous Statistical Inference.

[B14] Storey J (2002). A direct approach to false discovery rates. J R Statist Soc B.

[B15] Fisher RA (2007). Statistical Methods for Research Workers.

[B16] Hedges LV, Olkin I (1985). Statistical Methods for Meta-analysis.

[B17] Shi L, Reid LH, Jones WD, Shippy R, Warrington JA, Baker SC (2006). The MicroArray Quality Control (MAQC) project shows inter- and intraplatform reproducibility of gene expression measurements. Nat Biotechnol.

[B18] Tan Y, Shi L, Tong W, Wang C (2005). Multi-class cancer classification by total principal component regression (TPCR) using microarray gene expression data 5. Nucleic Acids Res.

[B19] Kanehisa M, Goto S, Hattori M, oki-Kinoshita KF, Itoh M, Kawashima S (2006). From genomics to chemical genomics: new developments in KEGG. Nucleic Acids Res.

[B20] Kanehisa M, Goto S (2000). KEGG: kyoto encyclopedia of genes and genomes. Nucleic Acids Res.

[B21] Kanehisa M (1997). A database for post-genome analysis. Trends Genet.

[B22] Dennis G, Sherman BT, Hosack DA, Yang J, Gao W, Lane HC, Lempicki RA (2003). DAVID: Database for Annotation, Visualization, and Integrated Discovery. Genome Biol.

[B23] Bozcaarmutlu A, Arinc E (2007). Effect of mercury, cadmium, nickel, chromium and zinc on kinetic properties of NADPH-cytochrome P450 reductase purified from leaping mullet (Liza saliens). Toxicol In Vitro.

[B24] Plewka A, Plewka D, Nowaczyk G, Brzoska MM, Kaminski M, Moniuszko-Jakoniuk J (2004). Effects of chronic exposure to cadmium on renal cytochrome P450-dependent monooxygenase system in rats. Arch Toxicol.

[B25] Zaccaro MC, Salazar C, Zulpa dC, Storni dC, Stella AM (2001). Lead toxicity in cyanobacterial porphyrin metabolism. Environ Toxicol.

[B26] Komatsu M, Furukawa T, Ikeda R, Takumi S, Nong Q, Aoyama K (2007). Involvement of mitogen-activated protein kinase signaling pathways in microcystin-LR-induced apoptosis after its selective uptake mediated by OATP1B1 and OATP1B3. Toxicol Sci.

[B27] Efron B, Tibshirani R (1993). An introduction to the bootstrap.

[B28] Dudoit S, Yang Y, Matthew J, Speed TP (2000). Statistical methods for identifying differentially expressed genes in replicated cDNA microarray experiments.

